# Computerised analysis of facial emotion expression in eating disorders

**DOI:** 10.1371/journal.pone.0178972

**Published:** 2017-06-02

**Authors:** Jenni Leppanen, Marcela Marin Dapelo, Helen Davies, Katie Lang, Janet Treasure, Kate Tchanturia

**Affiliations:** 1 Department of Psychological Medicine, Institute of Psychology, Psychiatry, and Neuroscience, King’s College London, London, United Kingdom; 2 Illia State University, Department of Psychology, Tbilisi, Georgia; Universitatsklinikum Tubingen, GERMANY

## Abstract

**Background:**

Problems with social-emotional processing are known to be an important contributor to the development and maintenance of eating disorders (EDs). Diminished facial communication of emotion has been frequently reported in individuals with anorexia nervosa (AN). Less is known about facial expressivity in bulimia nervosa (BN) and in people who have recovered from AN (RecAN). This study aimed to pilot the use of computerised facial expression analysis software to investigate emotion expression across the ED spectrum and recovery in a large sample of participants.

**Method:**

297 participants with AN, BN, RecAN, and healthy controls were recruited. Participants watched film clips designed to elicit happy or sad emotions, and facial expressions were then analysed using FaceReader.

**Results:**

The finding mirrored those from previous work showing that healthy control and RecAN participants expressed significantly more positive emotions during the positive clip compared to the AN group. There were no differences in emotion expression during the sad film clip.

**Discussion:**

These findings support the use of computerised methods to analyse emotion expression in EDs. The findings also demonstrate that reduced positive emotion expression is likely to be associated with the acute stage of AN illness, with individuals with BN showing an intermediate profile.

## Introduction

Facial expressions are part of body language and important social signals, which play a crucial role in social communication and interaction [1]. Communicating positive emotion contributes to social relationships, signalling social integration, and facilitating cooperation and affiliation [1]. Expressing negative emotions can also have a positive impact on the maintenance of social relationships, through increasing intimacy in relationships and eliciting others’ willingness to offer help and support [2]. Moreover, expression of negative emotions may have subjective benefits, reducing physiological arousal during exposure to negative emotional stimuli [3].

Difficulties in social-emotional communication have been documented in eating disorders (ED) and are believed to contribute to the development and maintenance of disordered eating [4]. Specifically, it has been proposed that individuals with anorexia nervosa (AN) perceive emotions as a threat [5], and behaviours such as dietary restriction or excessive exercise may help them to avoid distressing emotions and to provide a sense of control [6, 7]. Thus, the ED would function as a maladaptive emotion regulation strategy numbing the expression of emotions, which leads to social isolation and family conflicts [4, 8]. Despite lack of theoretical framework, based on behavioural findings similar maladaptive coping mechanisms are likely to be present in other forms of ED.

A recent meta-analysis found that relative to healthy individuals, people with AN displayed less positive facial affect with a large effect size in response to a variety of positive stimuli, ranging from pure emotion to short clips extracted from famous films [9]. Similarly, relative to healthy individuals, people with AN also displayed less congruent facial affect with a medium effect size in response to negative emotional stimuli [9]. Still, the studies included in the meta-analyses did not report significant differences between healthy control (HC) and AN groups in subjective positive and negative affect reported after exposure to the emotional stimuli [10–13]. Thus, there appears to be converging evidence demonstrating that people with AN have reduced facial expressivity of emotions while viewing emotionally provoking stimuli [9].

Emotion expression has been less studied in people with bulimia nervosa (BN) [11, 14, 15]. For example, Tárrega, Fagundo (15) have investigated emotional facial affect in response to a therapeutic video game, designed to target emotion regulation in general irrespective of valence [15]. Results reported that the BN group displayed significantly more negative facial affect than HC participants during the game [15]. Another similar study investigating spontaneous facial expressions in patients with BN and binge eating disorder during the same computer game, found that the patients expressed significantly less positive and negative facial affect than HCs [16]. Other studies investigating expression of positive and negative emotion in people with BN have found no differences between BN and HC participants in spontaneous positive and negative facial expressions in response to emotional film clips or during verbal expression of emotions when asked to talk about positive and negative emotional experiences [11, 14]. Together, these findings suggest that there is currently little certainty about anomalies in facial expressivity in BN.

Even fewer studies thus far have investigated expression of facial affect among people who have recovered from AN. These studies have reported no significant differences between recovered and HC participants in expression of positive or negative affect in response to emotionally provoking film clips [17, 18]. However, further research is required to gain a clear understanding of emotion expression in different forms of ED and different stages of recovery.

Most of the studies in eating disorders have examined emotion expression utilising manual coding systems, such as the Facial Expression Coding System [19]. However, such manual coding methods require extensive training, are labour-intensive, time consuming and potentially problematic for interrater reliability [19, 20]. Additionally, manual coding can be confounded by human error and bias. The use of more physiological techniques such as facial electromyography (EMG), on the other hand, can be obstructive as it requires electrodes to be placed on the participants’ skin. Furthermore, as facial EMG records electrical signal from muscle fibres it can be confounded by muscle movements that are not related to emotional facial expressions arising from speech, muscle fatigue, or other activities and responses [21–23]. Using computerised automated facial affect recognition software that can classify facial expressions from still images and video and provide information about the intensity of the emotion expressed can be methodologically and ecologically more sound and robust [24]. To our knowledge few studies have thus far employed such technology to investigate anomalies in emotion expression in ED.

The aim of the current study was to pool together data from previous studies [11, 12, 17, 25, 26] as well as new and unpublished data in order to create a large dataset relating to emotion expression in people with ED across the diagnostic spectrum and stage of recovery. With this large dataset, we aimed to pilot the use of a computerised facial expression analysis software, the FaceReader, in ED. Based on previous work outlined above, we hypothesised that people with current ED would display less facial affect while viewing emotionally provoking stimuli compared to HC participants and people recovered from AN.

## Method

### Participants

Two hundred and ninety-seven participants were recruited between 2009 and 2016. The ED participants included 100 people with AN and 33 people with BN. The patient group was recruited from the South London and Maudsley NHS Foundation Trust clinical services and through adverts posted on ED charity websites (Beat and Succeed) and a university campus. All ED participants had been diagnosed by clinicians and met the diagnostic criteria outlined in the DSM-5. Diagnoses were confirmed with the research version of the Structured Clinical Interview for DSM-5 [27]. Thirty-eight people recovered from AN (RecAN) and 126 HC participants were recruited from the community through adverts posted on ED charity websites (Beat and Succeed) and amongst King’s College London students and staff, and the local community. Recovery was defined as having had a past diagnosis of AN, currently having a healthy BMI (18.5–25.0) and not reporting eating disorder symptoms. Participants were excluded from the study if they were male, were diagnosed with neurological disorders, or had a history of or current alcohol or drug abuse. Additionally, HC participants were excluded if they had a history of, or a current diagnosis of, a psychiatric disorder or were taking regular medication. A total of 55% of the AN participants, 25% of the BN participants, and 32% of the RecAN participants were taking psychotropic medication. All participants gave written informed consent before taking part and all procedures were conducted in accordance with the latest version of the Declaration of Helsinki and were approved by the Camberwell St Giles NRES, Oxfordshire NRES, West London NRES, London Dulwich NRES, and South London and Maudsley NHS research ethics committees (Ethical approval numbers: 13/LO/0201; 08/H0606/58; 12/LO/2015; 14/LO/0128).

### Self-report questionnaire measures

The Eating Disorders Examination Questionnaire (EDEQ) is a 36-item self-report assessment of eating disorder psychopathology [28]. In addition to a global score the EDEQ also provides an assessment of restraint, eating concern, weight concern, and shape concern over the past 28 days.

The studies from which the data was acquired used two different self-report measures to assess depression and anxiety: the Hospital, Anxiety and Depression Scale (HADS) and the Depression, Anxiety and Stress Scale (DASS). The HADS is a 14-items self-report assessment of feelings of anxiety and depression experienced over the past over the past week [29]. The DASS is a 21-item self-report assessment of depression, anxiety, and stress experienced over the past week [30].

### The film task

During the film task participants were presented with two video clips lasting 2–2.5 minutes each, during which time they were filmed with a small video camera. The first film clip, Film 1, featured a humorous scene from the film *Four weddings and a funeral* (1994) and was used to evoke positive emotions. The second film clip, Film 2, featured a sad scene from the film *Shadowlands* (1999) and was used to evoke negative emotions, particularly sadness. A manipulation check was conducted as a part of the statistical analysis, to ensure the film clips elicited the intended emotions as opposed to the opposite emotions. The two film clips were presented in a fixed order. To reduce carry-over effects from one film clip to the other, the clips were separated by a brief clip featuring computer generated waves lasting 30 seconds (for more details Davies, Schmidt (25)).

### Noldus FaceReader

The Noldus FaceReader (Noldus Information Technology b.v., www.noldus.com) is a facial analysis program, which detects emotional facial expressions in film and photographs. The FaceReader can detect six basic emotions, happiness, sadness, anger, fear, surprise, and disgust, as well as neutral states. The FaceReader can also analyse valence of facial expressions as well as general state of arousal. The video stimuli are analysed frame-by-frame detecting the intensity to which each of the six basic emotions are expressed on a scale from 0 to 1, where 0 indicates the emotion is not present and 1 indicates maximum intensity. In the current study we focused on emotions the film clips were designed to elicit, happiness and sadness.

Prior to analysis, all videos were calibrated to each participant’s own neutral expression to achieve the most accurate results. The videos were then analysed using the batch analysis mode, which analyses each video sequentially without need for supervision.

### Data source

The video recordings were pooled from datasets of previously published studies [11, 12, 17, 25, 26]. In addition to including published data, previously unpublished data from these studies was also included. The unpublished data constituted 43% of the present dataset. This data was not published as part of the above studies for a variety of reasons, including having been only recently collected and not meeting sample size requirements alone.

### Statistical analysis

All data was analysed with Stata 14 (Stata Corp.). Group differences in questionnaire data were investigated with non-parametric median chi-squared test. Prior to analysis, the raw anxiety and depression scores from DASS and HADS questionnaires were converted into standardised z-scores. Significance was set at p < 0.05.

Prior to statistical analysis all facial expression proportion data was transformed out of the fixed range between 0 and 1 with the arcsine transformation to allow further analysis. Facial expression data was then entered into mixed effects linear model with 1000 bootstrap repetitions using the *mixed* command. Group (HC, AN, BN, RecAN) and film (Film 1, Film 2) were entered as fixed effects with a random intercept. Significant effects and interactions were further explored with post-hoc contrasts and pairwise comparisons. Confidence intervals in the post-hoc pairwise comparisons were adjusted for multiple comparisons with the Bonferroni method using the *mcompare(bonferroni)* command.

Additionally, relationships between expressions of happiness and sadness, and BMI, eating disorder psychopathology as measured on the EDEQ, anxiety, and depression were investigated with the non-parametric spearman’s rho correlation. Confidence intervals in the correlation analyses were adjusted for multiple comparisons with the Bonferroni method using the *bonferroni* command.

## Results

### Clinical and demographic characteristics

The sample clinical and demographic characteristics are presented in Table 1. As expected, there was a significant difference between the groups in BMI, ED psychopathology, anxiety, and depression.

**Table 1:** Sample clinical and demographic characteristics.

	AN (N = 100) Median [Q1,Q3]	BN (N = 33) Median [Q1,Q3]	RecAN (N = 38) Median [Q1,Q3]	HC (N = 126) Median [Q1,Q3]	Group differences: Χ^2^ statistic, p value
BMI	15.02 [13.5, 17.02]	20.00 [19.00, 21.10]	19.90 [19.00, 21.70]	21.45 [20.20, 23.81]	AN vs. BN: Χ^2^ = 23.87, p < 0.001 AN vs. RecAN: Χ^2^ = 33.18, p < 0.001 AN vs. HC:Χ^2^ = 135.22, p < 0.001 BN vs. RecAN: Χ^2^ = 0.01, p = 0.920 BN vs. HC: Χ^2^ = 4.13, p = 0.042 RecAN vs. HC: Χ^2^ = 1.57, p = 0.210
EDEQ total	4.19 [3.16, 5.00]	4.45 [2.88, 5.34]	1.11 [0.63, 2.04]	0.60 [0.22, 1.02]	AN vs. BN: Χ^2^ = 0.19, p = 0.663 AN vs. RecAN: Χ^2^ = 26.28, p < 0.001 AN vs. HC: Χ^2^ = 116.56, p < 0.001 BN vs. RecAN: Χ^2^ = 19.57, p < 0.001 BN vs. HC: Χ^2^ = 32.97, p < 0.001 RecAN vs. HC: Χ^2^ = 9.58, p = 0.002
Anxiety	0.66 [0.23, 1.64]	0.39 [-0.09, 0.84]	-0.39 [-0.77, 0.10]	-0.80 [-1.01, -0.60]	AN vs. BN: Χ^2^ = 2.12, p = 0.145 AN vs. RecAN: Χ^2^ = 17.45, p < 0.001 AN vs. HC: Χ^2^ = 116.97, p < 0.001 BN vs. RecAN: Χ^2^ = 9.08, p = 0.003 BN vs. HC: Χ^2^ = 36.80, p < 0.001 RecAN vs. HC: Χ^2^ = 13.24, p < 0.001
Depression	0.79 [0.23, 1.37]	0.42 [0.01, 1.03]	-0.52 [-0.70, -0.33]	-0.70 [-0.90, -0.56]	AN vs. BN: Χ^2^ = 2.25, p = 0.134 AN vs. RecAN: Χ^2^ = 21.85, p < 0.001 AN vs. HC: Χ^2^ = 126.28, p < 0.001 BN vs. RecAN: Χ^2^ = 18.04, p < 0.001 BN vs. HC: Χ^2^ = 35.11, p < 0.001 RecAN vs. HC: Χ^2^ = 8.83, p = 0.003

AN = anorexia nervosa; BN = bulimia nervosa; REC = recovered from anorexia nervosa; EDEQ = Eating Disorder Examination Questionnaire

### Expressions of happiness

**Fig 1:**
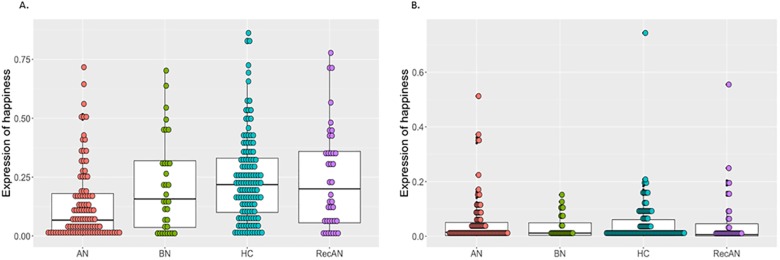
The intensity of expressions of happiness during Film 1 and Film 2. A: The intensity of expressions of happiness during Film 1; B. The intensity of expressions of happiness during Film 2. The box plot represents summary statistic, highlighting the median and interquartile range with minimum and maximum. The grey dots represent each individual data point. AN = anorexia nervosa, BN = bulimia nervosa, RecAN = recovered from anorexia nervosa, HC = healthy comparison.

The intensity of expressions of happiness during Film 1 and Film 2 are presented in Fig 1. The manipulation check confirmed that all participants expressed significantly more happiness in response to Film 1 than Film 2 (Table 2).

**Table 2:** The intensity of expressions of happiness and sadness during Film 1 and Film 2 within the AN, BN, RecAN, and HC groups.

Emotion	Film clip	AN (N = 100) Mean (SD)	BN (N = 33) Mean (SD)	RecAN (N = 38) Mean (SD)	HC (N = 126) Mean (SD)	Χ^2^ statistic, p value
Happiness	Film 1	0.13 (0.16)	0.21 (0.20)	0.25 (0.22)	0.24 (0.19)	Film: Χ^2^ = 218.39, p < 0.001 Group: Χ^2^ = 27.98, p < 0.001 Film x group: Χ^2^ = 26.63, p < 0.001
	Film 2	0.05 (0.09)	0.03 (0.04)	0.05 (0.10)	0.04 (0.08)	
Sadness	Film 1	0.02 (0.07)	0.04 (0.07)	0.01 (0.03)	0.03 (0.06)	Film: Χ^2^ = 38.29, p < 0.001 Group: Χ^2^ = 3.65, p = 0.302 Film x group: Χ^2^ = 0.02, p = 0.999
	Film 2	0.04 (0.08)	0.06 (0.13)	0.05 (0.08)	0.06 (0.13)	

AN = anorexia nervosa; BN = bulimia nervosa; RecAN = recovered from anorexia nervosa; HC = healthy comparison; Film 1 = humorous film clip; Film 2 = sad film clip

There was also a significant effect of group and a significant film by group interaction (Table 2). The interaction was further explored by investigating differences between groups within each film condition. There was a significant difference between groups in expressions of happiness only during Film 1 (Χ^2^ = 42.95, p < 0.001), but not during Film 2 (Χ^2^ = 0.63, p = 0.889). Pairwise comparison revealed that HC participants expressed significantly more happiness than AN (Z = 6.28, p < 0.001, 95% CI [0.09, 0.23]) during Film 1. Similarly, the RecAN participants expressed significantly more positive affect than AN participants during Film 1 (Z = 3.95, p < 0.001, 95% CI [0.05, 0.25]). The difference between BN and AN groups (Z = 2.46, p = 0.082, 95% CI [-0.01, 0.22]), approached significance. There were no significant differences between HC and BN group (Z = 1.38, p = 1.000, 95% CI [-0.05, 0.17]), HC and RecAN participants (Z = 0.34, p = 1.000, 95% CI [-0.09, 0.11]) or RecAN and BN participants (Z = 0.89, p = 1.000, 95% CI [-0.09, 0.18]) in expressions of happiness while viewing Film 1. The confidence intervals were adjusted for multiple comparisons using the Bonferroni method.

### Expressions of sadness

**Fig 2:**
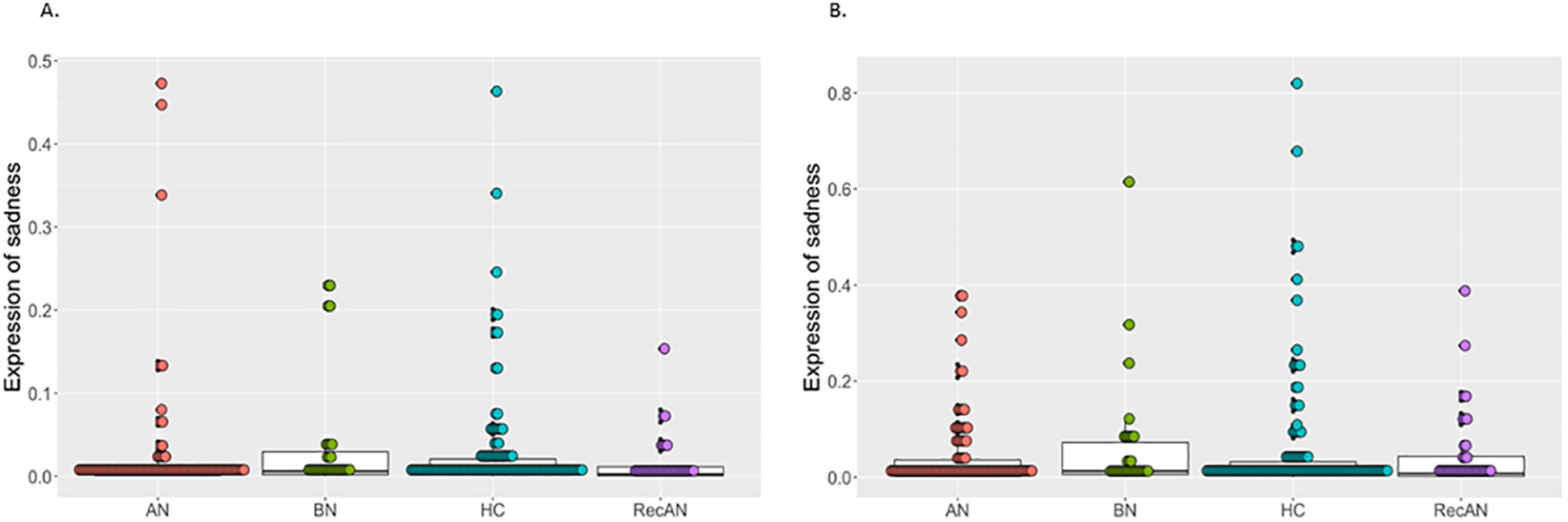
The intensity of expressions of sadness during Film 1 and Film 2. A. The intensity of expressions of sadness during Film 1; B. The intensity of expressions of sadness during Film 2. The box plot represents summary statistic, highlighting the median and interquartile range with minimum and maximum. The grey dots represent each individual data point. AN = anorexia nervosa, BN = bulimia nervosa, RecAN = recovered from anorexia nervosa, HC = healthy comparison.

The intensity of expressions of sadness during Film 1 and Film 2 are presented in Fig 2. The manipulation check confirmed that all participants expressed more sadness while viewing Film 2 than Film 1 (Table 2). There were no other significant effects of interactions.

### Correlations between facial expressions and clinical variables

Correlations between expressions of happiness and sadness, and BMI, eating disorder psychopathology, anxiety, and depression are presented in S1 Table. The confidence intervals were adjusted for multiple comparisons using the Bonferroni method. The analysis revealed no significant correlations.

## Discussion

The aim of the present study was to pilot the use of automated facial expression analysis software in a large group of people with ED and HC participants. The present study showed that relative to HC and RecAN participants, people with AN displayed less positive facial affect, happiness, in response to the humorous film clip. Additionally, there were trend level differences between the BN and AN participants in expression of positive affect during the humorous film clip. There were no significant group differences in facial expressivity while viewing the sad film. Finally, the present findings show that automated facial expression analysis software is a useful tool investigating expressions in facial affect in healthy and in clinical populations.

The findings from the automated facial expression analysis are in line with previous work showing that relative to RecAN and HC participants, people with AN show attenuation of positive facial affect in response to a variety of emotionally provoking stimuli [9]. Additionally, the present results also replicate previous findings that reduced facial expressivity may be part of acute illness and possibly exaggerated by starvation [17, 18]. One possible interpretation of these findings is that reduced expression in the acute stage of illness is part of maladaptive emotion regulation based on avoidance and suppression. Indeed, relative to healthy individuals, people with acute AN report greater tendency to rely on emotion avoidance and suppression [6, 31]. Furthermore, a recent systematic review found that people recovered from AN were similar to HCs, reporting less emotion avoidance and suppression than those with acute AN [31].

Another possible interpretation is that people with acute AN may express less positive affect due to reduced interest and desire to seek positive emotional experiences [32–34]. Previous studies have found that relative to HCs, people with acute AN report more social anhedonia, which correlated highly with eating disorder psychopathology, and elevated incidence of autistic symptoms [32, 33, 35, 36]. Qualitative studies have also found that people with AN report limited friendship networks and poor friendship quality [37, 38]. Additionally, behavioural studies have found that people with acute AN show attentional avoidance of positive emotional stimuli, and report low perceived social rank and increased external shame [39–41]. Interestingly, a recent systematic review found that people who have recovered from AN report increased desire to seek pleasure in social interactions and relationships, and less submissiveness and tendency to engage in negative social comparisons [31]. Thus, people in the acute stage of AN may have reduced desire to engage with positive emotionally provoking stimuli leading to reduced expression of positive facial affect.

The present findings showed that people with BN displayed marginally more positive facial affect than the people with AN, but were not significantly different from the HCs. These findings are some way supported by recent systematic reviews, which have reported that both AN and BN were characterised by emotion dysregulation based on emotion and situation avoidance [31, 42]. However, BN has also been suggested to be characterised by elevated sensation seeking, urgency, and difficulties in behavioural inhibition during times of distress [42, 43]. Additionally, a large scale cohort study found that BN diagnosis and bulimic symptoms clustered together with mid-to-high incidence of novelty seeking [44]. Interestingly, a recent study investigating spontaneous facial expression and personality traits in BN and binge eating disorder, found increased positive facial expression was positive associated with novelty and reward seeking, while increased expression on anger was negatively associated with self-directedness [16]. However, it is of note that in the present study the BN participants were not significantly different from the HCs. Thus, taken together these findings suggest that BN may be characterised by an intermediate profile, but replication of these findings with a large sample of people with BN is of importance before firm conclusions are drawn.

Finally, the present study did not find significant differences between the groups in emotion expression while viewing Film 2. These findings go some way to replicate findings from previous work showing no significant differences in frequency of negative facial expressions in response to different, but similarly sad stimuli between people with ED and HC participants [45]. Nevertheless, these findings are in direct contrast with findings from other previous studies [13], suggesting the further exploration of expressions of negative affect in ED would be of interest.

### Clinical implications

The present findings add to the wealth of evidence that people with AN show reduced expression of positive facial affect. Reduced expression of facial affect can have important emotional and social consequences [3, 46]. One study found that those who were suppressing their emotional responses reported elevated negative affect and had higher blood pressure than those expressing their emotions [3]. Moreover, a recent behavioural study found that people who were displaying blunted response to positive emotional stimuli were judged more negatively by the other participants and increased the other participants’ desire to avoid these individuals [46]. Taken together, the elevated negative mood and social anhedonia, poor social relationships, and isolation reported by people with ED may be in some way related to blunted facial expressivity [33, 38].

Thus, the present findings highlight the need for interventions that target emotion expression in ED, particularly those aimed at increasing the expression of positive affect. These findings may also be useful as psychoeducational material for patients and families, who may struggle with reduced facial expression in the patient with ED [47]. Additionally, new therapeutic approaches and family based interventions, which increasingly build on positive psychology and experimental psychology (e.g. awareness of facial feedback hypothesis, still face paradigm and other experimental findings), could benefit from taking these findings into account. Current innovative approaches in emotion coaching facilitate cognitive awareness of poor social signalling via psychoeducation, experiential exercises, role plays and show promise [48]. However, these therapeutic developments are still work in progress and there is clear need for larger clinical trials [48].

The present findings also demonstrate that automated facial expressions analysis software can be a useful addition in clinical research (e.g. evaluation of outcomes after emotion skills training). It allows faster, more accurate, and less labour-intensive analysis of expression of facial affect than manualised versions. Many of the currently available software, including the FaceReader, can also be used to conduct live analysis. Live analysis may be a useful addition to provide biofeedback in therapeutic interventions targeting facial expressivity.

### Limitations

Regardless of our effort to pull large sample for analysis the main limitation of the present study was the relatively small RecAN and BN sample sizes, this also reflects general trend in the literature. In order to gain a better understanding of alterations in emotion expression in ED, future research may benefit from further investigating expression of facial affect in BN, in different stages of recovery, and in people who have recovered from ED.

Additionally, the present study focused only on expression of positive affect and sadness. Previous work has shown that people with ED may have elevated subjective feelings of hostility, anger, and disgust [31]. Future research may benefit from further exploration of the expression of these emotions in ED.

Finally, the use of automated facial expressions analysis software, such as the FaceReader, is not without limitations. To gain reliable results the participant should look frontally or pseudo-frontally to the camera [49]. Additionally, hats, glasses, certain hairstyles that cover the faces, heavy facial hair, or anything else that can partially cover the face can cause problems with accurate classification [49]. Further, good lighting conditions, ideally frontal lighting, is essential for accurate classification [49]. Any shadows and reflections that partially cover the faces can cause problems [49].

## Conclusion

The current study examined facial expressivity in response to positive and negative emotional film clips in a large dataset of ED, RecAN, and HC participants using computerised automated facial expression analysis software. The findings revealed that computerised facial expressions analysis software to be useful addition to clinical research. Additionally, the findings add to the steady accumulation of evidence that people with AN show less positive facial affect than healthy individuals and people recovered from AN. Thus, these findings suggest that in AN reduced facial affect is associated with acute stage of illness and may play a role in fuelling the illness by increasing negative affect and social isolation. People with BN, on the other hand, appear to have an intermediate profile. However, further work is required to elucidate the atypical facial expression in BN.

## Supporting information

S1 Dataset Raw emotion expression data. (CSV)

S1 Table Correlations between expressions of happiness and sadness, and psychopathology within the AN, BN, and REC groups. AN = anorexia nervosa; BN = bulimia nervosa; REC = recovered from anorexia nervosa; EDEQ = Eating Disorder Examination Questionnaire; Film 1 = humorous film clip; Film 2 = sad film clip
(DOCX)
