# Supplementary material for: Computerised analysis of facial emotion expression in eating disorders
Source: PLoS One. 2017 Jun 2;12(6):e0178972. doi: 10.1371/journal.pone.0178972 (PMC5456367; doi:10.1371/journal.pone.0178972)
Supplement: S1 Table — AN = anorexia nervosa; BN = bulimia nervosa; REC = recovered from anorexia nervosa; EDEQ = Eating Disorder Examination Questionnaire; Film 1 = humorous film clip; Film 2 = sad film clip (DOCX) [file pone.0178972.s002.docx]

S1 Table. Correlations between expressions of happiness and sadness, and psychopathology within the AN, BN, and REC groups

| Film clip | Group | Emotion | EDEQ Global | Anxiety | Depression | BMI |
| --- | --- | --- | --- | --- | --- | --- |
| Film 1 | AN | Happiness | ρ = -0.05, p = 1.000 | ρ = -0.03, p = 1.000 | ρ = -0.08, p = 1.000 | ρ = -0.08, p = 1.000 |
|  |  | Sadness | ρ = 0.03, p = 1.000 | ρ = -0.06, p = 1.000 | ρ = 0.13, p = 1.000 | ρ = -0.17, p = 1.000 |
|  | BN | Happiness | ρ = 0.22, p = 1.000 | ρ = 0.07, p = 1.000 | ρ = -0.11, p = 1.000 | ρ = -0.23, p = 1.000 |
|  |  | Sadness | ρ = -0.09, p = 1.000 | ρ = 0.22, p = 1.000 | ρ = -0.05, p = 1.000 | ρ = 0.42, p 0.840 |
|  | REC | Happiness | ρ = -0.22, p = 1.000 | ρ = 0.23, p = 1.000 | ρ = -0.17, p = 1.000 | ρ = -0.12, p = 1.000 |
|  |  | Sadness | ρ = 0.34, p = 1.000 | ρ = -0.25, p = 1.000 | ρ = 0.28, p = 1.000 | ρ = 0.20, p = 1.000 |
| Film 2 | AN | Happiness | ρ = 0.07, p = 1.000 | ρ = -0.09, p = 1.000 | ρ = 0.07, p = 1.000 | ρ = -0.24, p = 1.000 |
|  |  | Sadness | ρ = 0.05, p = 1.000 | ρ = -0.11, p = 1.000 | ρ = 0.14, p = 1.000 | ρ = 0.002, p = 1.000 |
|  | BN | Happiness | ρ = 0.14, p = 1.000 | ρ = 0.17, p = 1.000 | ρ = -0.04, p = 1.000 | ρ = 0.09, p = 1.000 |
|  |  | Sadness | ρ = -0.21, p = 1.000 | ρ = 0.11, p = 1.000 | ρ = -0.11, p = 1.000 | ρ = -0.03, p = 1.000 |
|  | REC | Happiness | ρ = -0.28, p = 1.000 | ρ = -0.19, p = 1.000 | ρ = -0.20, p = 1.000 | ρ = 0.05, p = 1.000 |
|  |  | Sadness | ρ = 0.25, p = 1.000 | ρ = -0.01, p = 1.000 | ρ = -0.08, p = 1.000 | ρ = 0.03, p = 1.000 |

AN = anorexia nervosa; BN = bulimia nervosa; REC = recovered from anorexia nervosa; EDEQ = Eating Disorder Examination Questionnaire; Film 1 = humorous film clip; Film 2 = sad film clip
